# Tacrolimus versus cyclophosphamide for patients with idiopathic membranous nephropathy and treated with steroids: a systematic review and meta-analysis of randomized controlled trials

**DOI:** 10.1080/0886022X.2021.1914655

**Published:** 2021-05-21

**Authors:** Haiting Huang, Zhao Liang, Xintong Zheng, Qin Qing, Xiuri Du, Zhiming Tang, Meili Wei, Chen Wang, Qiuhong Zhong, Xu Lin

**Affiliations:** a Department of Nephrology, The Affiliated Hospital of Youjiang Medical University for Nationalities, Baise, China; b Department of Ultrasound, The Affiliated Hospital of Youjiang Medical University for Nationalities, Baise, China

**Keywords:** Idiopathic membranous nephropathy, tacrolimus, cyclophosphamide, steroids, meta-analysis

## Abstract

**Background:**

The therapeutic effects of tacrolimus (TAC) versus cyclophosphamide (CTX) were not fully illustrated for patients with idiopathic membranous nephropathy (IMN).

**Methods:**

The PubMed, EmBase, Cochrane library, and CNKI were systematically searched throughout March 2020 for randomized controlled trials evaluating the therapeutic effects of TAC versus CTX for IMN patients treated with steroids. The pooled relative risks (RRs) and weighted mean differences (WMDs) with 95% confidence intervals (CIs) were calculated using the random-effects model.

**Results:**

Twelve trials recruited a total of 868 IMN patients were identified and contained in final meta-analysis. Patients in TAC group was associated with an increased incidence of overall remission (12 trials: 868 patients; RR: 1.21; 95% CI: 1.11–1.31; *p* < 0.001) and complete remission (12 trials: 868 patients; RR: 1.50; 95% CI: 1.25–1.80; *p* < 0.001). Moreover, we noted TAC therapy significantly reduced urinary protein excretion (9 trials: 567 patients; WMD: −1.06; 95%CI: −1.41 to −0.71; *p* < 0.001), and increased serum albumin (9 trials: 567 patients; WMD: 5.37; 95%CI: 2.97 to 7.77; *p* < 0.001) than CTX therapy. Furthermore, no significant difference between TAC and CTX for serum creatinine was detected (6 trials: 378 patients; WMD: 0.15; 95%CI: −3.46 to 3.75; *p* = 0.936). Finally, the risk of alopecia (*p* = 0.008), infection (*p* = 0.045), leukocytosis (*p* = 0.002), and elevated ALT/AST (*p* = 0.011) in TAC group was significantly lower than CTX group, whereas TAC was associated with an increased risk of tremor than CTX (*p* = 0.010).

**Conclusions:**

This study found IMN patients treated with TAC combined with steroids provides a better therapeutic effect and less adverse events than those treated with CTX combined with steroids, with moderate-certainty evidence.

## Introduction

Idiopathic membranous nephropathy (IMN) as an autoimmune glomerular disease accounts for the most common cause of nephrotic syndrome in adults [1]. Patients with IMN were characterized by the thickened glomerular basement membrane, and the deposition of immune complexes at glomerular basement membrane. The symptoms of IMN ranged from proteinuria to nephropathy syndrome comorbidity with heavy proteinuria, also including hypertension, renal insufficiency, and microscopic hematuria [2]. Study have already demonstrated nearly 30–40% of IMN patients could progress to end-stage renal disease [3]. Therefore, the treatment objective for IMN patients was to induce a lasting reduction in proteinuria.

Immunosuppressive agents have already identified for restricting IMN patients suffering from severe nephrotic syndrome owing to severe proteinuria was associated with poor outcome for patients with IMN [4–6]. Moreover, remission from proteinuria was associated with favorable outcome, irrespective for complete remission or partial remission [7–9]. Therefore, the effective treatment strategy should be applied for IMN patients to improve the proteinuria. Tacrolimus (TAC) as a calcineurin inhibitors could improve the deteriorating renal function for patients IMN [10]. The use of TAC combined with steroids compared with cyclophosphamide (CTX) combined with steroids for improving severe proteinuria and the incidence of remission have already investigated in numerous studies, whereas mostly studies with smaller number of patients and inadequate follow-up duration [11–22]. Therefore, we conducted a systematic review and meta-analysis of randomized controlled trials (RCTs) to assess the treatment effectiveness of TAC versus CTX for IMN patients undergoing steroid therapy.

## Methods

### Data sources, search strategy, and selection criteria

The current systematic review and meta-analysis was performed following the Preferred Reporting Items for Systematic Reviews and Meta-Analysis Statement [23]. Study compared the therapeutic effects between TAC plus steroids and CTX plus steroids for IMN patients was eligible in this study, without any restrictions for published language and status. We systematically searched the databases of PubMed, EmBase, Cochrane library, and CNKI through March 2020 for eligible study and used the following search terms: (tacrolimus OR FK506 OR TAC) AND (IMN OR membranous nephropathy OR membranous glomerulonephropathy OR IMN OR MN). The details of search strategy are summarized in Supplementary Material 1. Moreover, we also searched for ongoing RCTs on the website http://clinicaltrials.gov/ (US NIH) and the metaRegister of Controlled Trials, which have registered and completed but not yet published. The reference lists from identified studies were also reviewed manually to select any new eligible study met the inclusion criteria.

Study was included if the following inclusion criteria was met: (1) Patients: all of the patients diagnosed with IMN; (2) Intervention: TAC plus steroids; (3) Control: CTX plus steroids; (4) Outcomes: overall remission, complete remission, urinary protein excretion, serum albumin, serum creatinine, and potential adverse events; and (5) Study design: the study had to have RCT design. The study selection process was conducted by two reviewers (HH and LZ), and conflicts between reviewers was settled by group discussion.

### Data collection and quality assessment

The abstracted information from retrieved studies included: first author’s name, publication year, country, sample size, mean age, percentage male, disease status, intervention, control, follow-up duration, and reported outcomes. Study quality for included studies was assessed by using the Cochrane Collaboration risk of bias instrument, and each item was assigned as yes, no, or unclear [24]. Two reviewers independently conducted the data abstracted and quality assessment (HH and LZ), and inconsistencies between reviewers was settled by group discussion until a consensus was reached.

### Statistical analysis

The therapeutic effects between TAC and CTX on the incidence of overall remission and complete remission were assigned as binary variables and relative risks (RRs) with 95% confidence intervals (CIs) were calculated in each trial before data pooling. Similarly, TAC versus CTX on urinary protein excretion, serum albumin, and serum creatinine were assigned as weighted mean differences (WMDs) and 95%CI in individual trial. All of the pooled analyses were conducted using the random-effects model, which could consider the varies underlying included trials [25,26]. Both *I*^2^ and *Q* statistic were applied to assess the heterogeneity across included studies, and *I*^2^ > 50.0% or *p* < 0.10 was considered as significant heterogeneity [27,28]. Sensitivity analysis was used to assess the robustness of pooled conclusion by sequential excluding individual trial [29]. Subgroup analysis was conducted for each investigated outcomes according to follow-up duration. Publication bias for each outcome was assessed by using the funnel plot, Egger, and Begg tests [30,31]. The inspection level for pooled outcomes are two-sided, and *p* < 0.05 was regarded as a significant difference between TAC and CTX. STATA software (Version 10.0; StataCorp, Texas, USA) was applied to conduct all analyses in this study.

## Results

### Literature search

**Figure 1.**
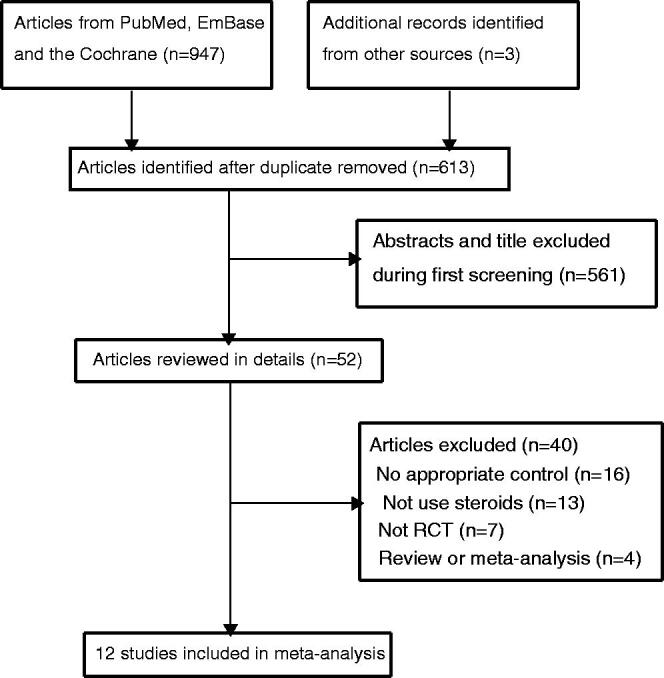
The PRISMA flowchart regarding the study selection process.

A total of 947 articles were identified by electronic searches, and 613 articles needed further title and abstracts evaluations after duplicate titles were excluded. Then 561 studies were excluded owing to irrelevant titles. The remaining 52 studies were retrieved for full-text evaluations, and 40 studies were excluded because of the following reasons: no appropriate control (*n* = 16); did not combined with steroids (*n* = 13), not RCTs (*n* = 7), review or meta-analysis (*n* = 4) (Supplementary Material 2). After this, 12 RCTs were selected for final meta-analysis (Figure 1). No new eligible study was observed by manually reviewing the reference lists from identified studies.

### Study characteristics

A total of 868 IMN patients from 12 RCTs were identified, and the baseline characteristics of identified studies and patients are summarized in Table 1. Eleven studies were conducted in China, and the remaining one study was conducted in India. The sample size of included studies ranged from 17 to 195, and the follow-up duration ranged from 6.0 to 24.0 months. Study quality was assessed using the Cochrane Collaboration risk of bias instrument, and the details in each trial are shown in Table 2.

**Table 1.** The characteristics of identified studies and enrolled patients.

Study	Publication year	Country	Sample size	Mean age (years)	Percentage male (%)	Disease status	Intervention	Control	Follow-up duration	Study quality
Chen et al. [11]	2009	China	17	16.0-60.0	NA	I–III IMN	TAC: 0.1 mg/ (kg·day), the whole blood trough level: 4–10 ng/mL; prednisone: 15–60 mg/day	CTX: 750 mg/m^2^; prednisone: 15–60 mg/day	6 months	2
Chen et al. [12]	2010	China	73	47.9	56.2	I–III IMN	TAC: 0.1 mg/(kg·day), the whole blood trough level: 5–10 ng/mL; prednisone: 1 mg/(kg·day)	CTX: 100 mg/day; prednisone: 1 mg/(kg·day)	12 months	5
Li et al. [13]	2012	China	30	53.2	NA	IMN	TAC: 0.07-0.1 mg/(kg·day), the whole blood trough level: 5–10 ng/mL; prednisone: 1 mg/(kg·day)	CTX: 750–1000 mg/m^2^; prednisone: 1 mg/(kg·day)	6 months	3
Xu et al. [14]	2013	China	100	57.0	60.8	I–IV IMN	TAC: 0.5-0.75 g/(m^2^·month), with a maximum dosage of 1.0 g/month; prednisone: 1 mg/(kg·day)	CTX: 500–750 mg/m^2^; prednisone: 1 mg/(kg·day)	18 months	4
He et al. [15]	2013	China	56	46.3	69.6	I–IV IMN	TAC: 1 mg/day or 2 mg/day, the whole blood trough level: 2–4 ng/mL; prednisone: 1 mg/ (kg·day)	CTX: 750 mg/m^2^; prednisone: 1 mg/(kg·day)	12 months	4
Peng et al. [16]	2016	China	60	42.4	55.0	IMN	TAC: 0.05 mg/(kg·day), the whole blood trough level: 4–8 ng/mL; prednisone: 0.5 mg/(kg·day)	CTX: 750 mg/m^2^; prednisone: 0.5 mg/(kg·day)	9 months	3
Ramachandran et al. [17]	2016	India	70	39.7	67.1	IMN	TAC: 0.1 mg/(kg·day); the whole blood trough level: 5–10 ng/mL; prednisolone: 0.5 mg/(kg·day)	CTX: 2 mg/(kg·day); methylprednisolone: 1 g/day; prednisolone 0.5 mg/(kg·day)	24 months	5
Ding et al. [18]	2016	China	195	50.3	49.7	IMN	TAC: 0.05–0.10 mg/(kg·day); the whole blood trough level: 5–10 ng/mL; prednisone: 1 mg/(kg·day)	CTX: 600-800 mg/m^2^; prednisone: 1.0 mg/(kg·day)	6 months	2
Zhang et al. [19]	2016	China	50	50.5	72.0	IMN	TAC: 0.1 mg/(kg·day); the whole blood trough level: 5–10 ng/mL; methylprednisolone: 12–16 mg/day	CTX: 600-1000 mg/m^2^; methylprednisolone: 0.8 mg/(kg·day)	6 months	3
Yang et al. [20]	2016	China	49	43.5	63.3	IMN	TAC: 30 mg/day; prednisone: 30 mg/day	CTX: 60 mg/day; prednisone: 30 mg/day	12 months	4
Abulitibu et al. [21]	2016	China	38	40.0	55.3	I–IV IMN	TAC: 0.05 mg/(kg·day); the whole blood trough level: 5–10 ng/mL; prednisone: 40 mg/day	CTX: 1000 mg/m^2^; prednisone: 40 mg/day	9 months	2
Tong et al. [22]	2019	China	130	53.6	48.5	IMN	TAC: 0.05–0.10 mg/(kg·day); the whole blood trough level: >5 ng/mL; prednisone: 0.5 mg/(kg·day)	CTX: 600–1000 mg/m^2^; prednisone: 0.5 mg/(kg·day)	6 months	2

**Table 2.** Risk of bias for individual study.

Study	Random sequence generation (selection bias)	Allocation concealment (selection bias)	Blinding of participants and personnel (performance bias)	Blinding of outcome assessment (detection bias)	Incomplete outcome data (attrition bias)	Selective reporting (reporting bias)	Other bias
Chen et al. [11]	Yes	No	No	Unclear	Yes	No	No
Chen et al. [12]	Yes	Yes	Yes	Yes	Yes	Unclear	Unclear
Li et al. [13]	Yes	No	No	Yes	Yes	No	No
Xu et al. [14]	Yes	Unclear	Unclear	Yes	Yes	Unclear	Unclear
He et al. [15]	Yes	Unclear	Unclear	Yes	Yes	Unclear	Unclear
Peng et al. [16]	Yes	No	No	Yes	Yes	No	No
Ramachandran et al. [17]	Yes	Yes	Yes	Yes	Yes	Unclear	Unclear
Ding et al. [18]	Yes	No	No	Unclear	Yes	No	No
Zhang et al. [19]	Yes	No	No	Yes	Yes	No	No
Yang et al. [20]	Yes	Unclear	Unclear	Yes	Yes	Unclear	Unclear
Abulitibu et al. [21]	Yes	No	No	Unclear	Yes	No	No
Tong et al. [22]	Yes	No	No	Unclear	Yes	No	No

### Overall remission and complete remission

**Figure 2.**
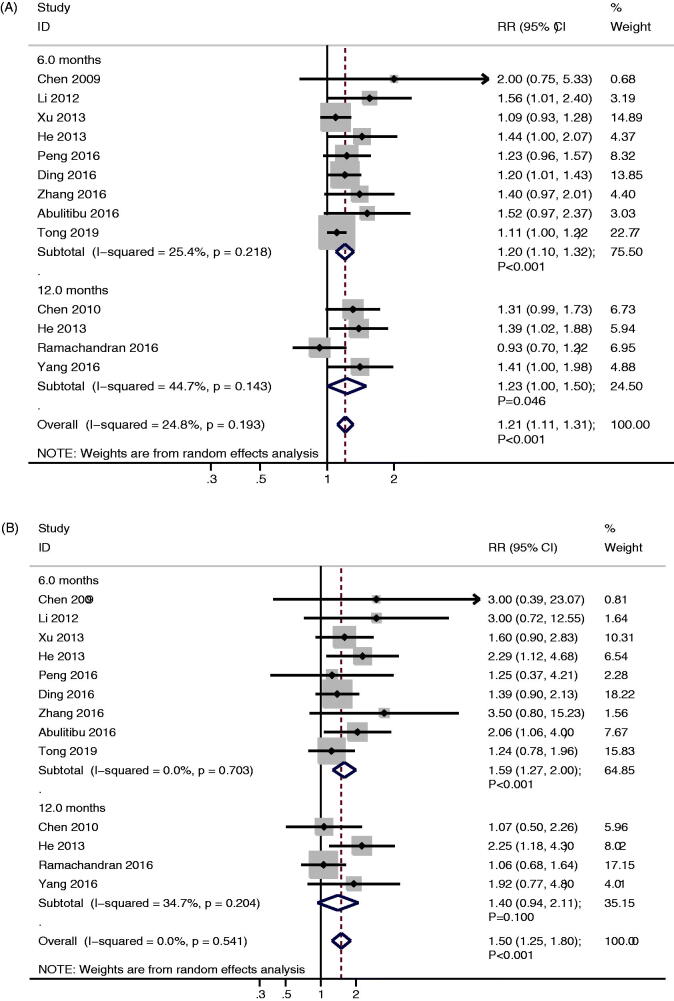
(A) The therapeutic effect of TAC versus CTX on the incidence of overall remission for IMN patients treated with steroids. (B) The therapeutic effect of TAC versus CTX on the incidence of complete remission for IMN patients treated with steroids.

All of the included studies reported the incidence of overall remission and complete remission between TAC and CTX. We noted TAC significantly increased the incidence of overall remission than CTX (RR: 1.21; 95%CI: 1.11–1.31; *p* < 0.001; Figure 2(A)), irrespective after 6.0 months follow-up (RR: 1.20; 95%CI: 1.10–1.32; *p* < 0.001) or 12.0 months follow-up (RR: 1.23; 95%CI: 1.00–1.50; *p* = 0.046). The heterogeneity among included studies was not associated with statistically significant (*I*^2^ = 24.8%; *p* = 0.193). Moreover, patients treated with TAC were associated with higher incidence of complete remission than those treated with CTX (RR: 1.50; 95%CI: 1.25–1.80; *p* < 0.001; Figure 2(B)). We noted the significant difference between TAC and CTX on the incidence of complete remission mainly seen after 6.0 months follow-up (RR: 1.59; 95%CI: 1.27–2.00; *p* < 0.001), while no significant difference between TAC and CTX for the incidence of complete remission after 12.0 months follow-up (RR: 1.40; 95%CI: 0.94–2.11; *p* = 0.100). The pooled conclusion for overall remission and complete remission were robustness and not altered by sequential excluding each individual trial (Supplementary Material 3). We noted potential significant publication bias for overall remission (*P* value for Egger: 0.003; *P* value for Begg: 0.001) and complete remission (*P* value for Egger: 0.027; *P* value for Begg: 0.200), while the conclusions were not changed after adjusted the publication bias using the trim and fill method (Supplementary Material 4) [32].

### Urinary protein excretion

**Figure 3.**
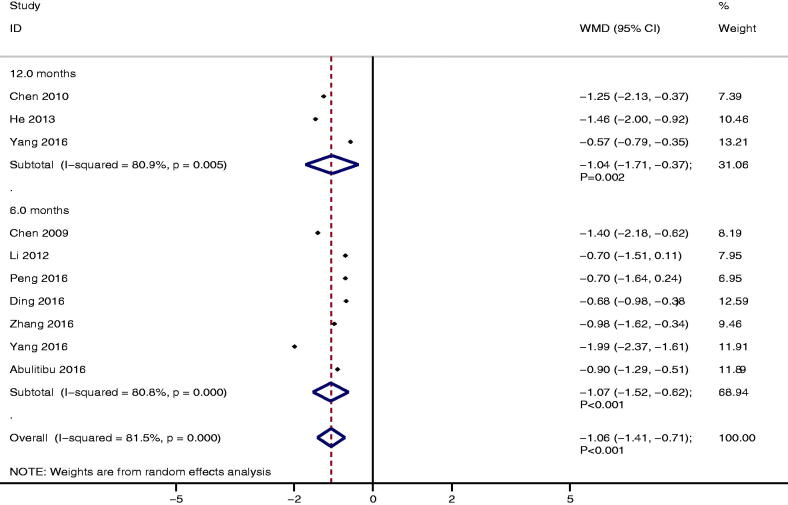
The therapeutic effect of TAC versus CTX on urinary protein excretion for IMN patients treated with steroids.

A total of 10 studies reported the level of urinary protein excretion between TAC and CTX. We noted TAC was associated with lower urinary protein excretion than CTX (WMD: −1.06; 95%CI: −1.41 to −0.71; *p* < 0.001; Figure 3), irrespective after 6.0 months follow-up (WMD: −1.07; 95%CI: −1.52 to −0.62; *p* < 0.001) or 12.0 months follow-up (WMD: −1.04; 95%CI: −1.71 to −0.37; *p* = 0.002). There was significant heterogeneity for urinary protein excretion (*I^2^* = 81.5%; *p* < 0.001). The conclusion was stable and not changed by a sensitivity analysis (Supplementary Material 3). No significant publication bias for urinary protein excretion was detected (*P* value for Egger: 0.260; *P* value for Begg: 0.721; Supplementary Material 4).

### Serum albumin

**Figure 4.**
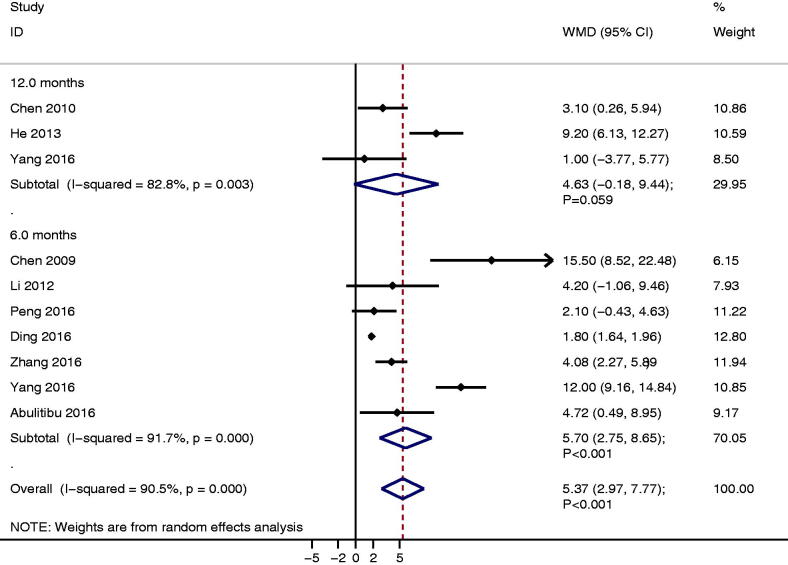
The therapeutic effect of TAC versus CTX on serum albumin for IMN patients treated with steroids.

A total of 10 studies reported the level of serum albumin between TAC and CTX. We noted TAC was associated with high serum albumin than CTX (WMD: 5.37; 95%CI: 2.97 to 7.77; *p* < 0.001; Figure 4), and significant heterogeneity was detected across included studies (*I^2^* = 90.5%; *p* < 0.001). Sensitivity analysis indicated the pooled conclusion was stable and not altered by sequential excluding each individual trial (Supplementary Material 3). Subgroup analysis found TAC significantly increased serum albumin after 6.0 months follow-up (WMD: 5.70; 95%CI: 2.75 to 8.65; *p* < 0.001), while no significant difference between TAC and CTX on serum albumin after 12.0 months (WMD: 4.63; 95%CI: −0.18 to 9.44; *p* = 0.059). Although the Begg test indicated no significant publication bias for serum albumin (*p* = 0.474), while the Egger test showed significant publication bias (*p* = 0.021; Supplementary Material 4). The conclusion was not altered after adjusted publication bias using the trim and fill method [32].

**Figure 5.**
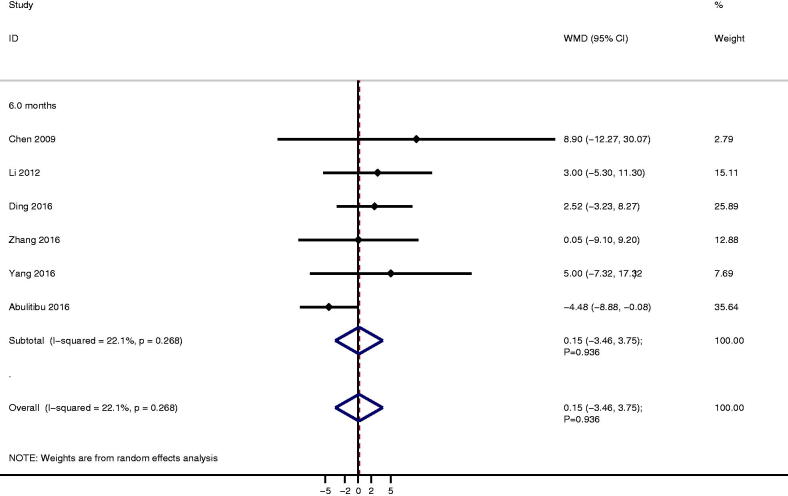
The therapeutic effect of TAC versus CTX on serum creatinine for IMN patients treated with steroids.

### Serum creatinine

A total of six studies reported the level of serum creatinine between TAC and CTX, and no significant difference between TAC and CTX for serum creatinine after 6.0 months follow-up (WMD: 0.15; 95%CI: −3.46 to 3.75; *p* = 0.936; Figure 5). Moreover, no significant heterogeneity was detected among included trials (*I*^2^ = 22.1%; *p* = 0.268). Sensitivity analysis indicated the conclusion was not changed after sequential excluding individual trial (Supplementary Material 3). There was no significant publication bias for serum creatinine (*P* value for Egger: 0.094; *P* value for Begg: 1.000; Supplementary Material 4).

### Adverse events

The pooled results for adverse events between TAC and CTX are summarized in Table 3. We noted TAC was associated with a reduced risk of alopecia (RR: 0.25; 95%CI: 0.09–0.70; *p* = 0.008), infection (RR: 0.53; 95%CI: 0.29–0.99; *p* = 0.045), leukocytosis (RR: 0.19; 95%CI: 0.07–0.54; *p* = 0.002), and elevated ALT/AST (RR: 0.46; 95%CI: 0.25–0.84; *p* = 0.011) than CTX. However, patients in TAC group were associated with an increased risk of tremor than those in CTX group (RR: 9.02; 95%CI: 1.71–47.62; *p* = 0.010). Moreover, there were no significant differences between TAC and CTX for the risk of elevated serum creatinine (*p* = 0.251), diarrhea (*p* = 0.470), gastrointestinal reaction (*p* = 0.064), glucose intolerance (*p* = 0.824), hypertension (*p* = 0.408), herpes zoster (*p* = 0.724), myelosuppression (*p* = 0.286), chemical cystitis (*p* = 0.347), embolism of deep vein (*p* = 0.529), gouty arthritis (*p* = 0.336), chest pain (*p* = 0.495), new-onset diabetes (*p* = 0.595), fracture (*p* = 0.497), and death (*p* = 0.496).

**Table 3.** The summary results for the incidence of adverse events.

Outcomes	Number of trials	RR and 95%CI	*P* value	Heterogeneity (%)	*P* value for heterogeneity
Elevated serum creatinine	3	2.87 (0.47–17.39)	0.251	0.0	0.998
Diarrhea	5	0.73 (0.31–1.73)	0.470	0.0	0.511
Gastrointestinal reaction	8	0.42 (0.17–1.05)	0.064	34.3	0.154
Alopecia	4	0.25 (0.09–0.70)	0.008	0.0	0.903
Infection	9	0.53 (0.29–0.99)	0.045	33.0	0.154
Leukocytosis	7	0.19 (0.07–0.54)	0.002	0.0	0.998
Elevated ALT/AST	8	0.46 (0.25–0.84)	0.011	0.0	0.885
Glucose intolerance	8	1.11 (0.44–2.78)	0.824	49.4	0.054
Tremor	3	9.02 (1.71–47.62)	0.010	0.0	0.936
Hypertension	5	1.55 (0.55–4.34)	0.408	0.0	0.555
Herpes zoster	4	1.30 (0.30–5.66)	0.724	0.0	0.448
Myelosuppression	1	0.20 (0.01–3.85)	0.286	—	—
Chemical cystitis	3	0.48 (0.10–2.22)	0.347	0.0	0.742
Embolism of deep vein	1	0.36 (0.02–8.64)	0.529	—	—
Gouty arthritis	3	1.69 (0.58–4.93)	0.336	0.0	0.931
Chest pain	1	3.00 (0.13–70.64)	0.495	—	—
New-onset diabetes	2	1.32 (0.47–3.71)	0.595	0.0	0.589
Fracture	1	3.00 (0.13–71.12)	0.497	—	—
Death	1	3.00 (0.13–70.83)	0.496	—	—

## Discussion

This study compared the therapeutic effects between TAC and CTX for IMN patients based on RCTs, and a total of 868 IMN patients across broad range of characteristics were identified from 12 RCTs. This study found TAC plus steroids was associated with an increased incidence of overall remission and complete remission than CTX plus steroids. Moreover, TAC plus steroids were associated with lower urinary protein excretion, and higher serum albumin as compared with CTX plus steroids. However, no significant difference between TAC and CT for serum creatinine was detected. All of pooled conclusions were robustness and the therapeutic effects between TAC and CTX could affect by follow-up duration. Finally, we noted TAC plus steroids were associated with lower risk of alopecia, infection, leukocytosis, and elevated ALT/AST, whereas the risk of tremor in TAC group was significantly increased.

Several systematic review and meta-analyses have already investigated the treatment effectiveness between TAC and CTX for patients with IMN. A meta-analysis conducted by Li et al. included eight RCTs and found TAC-based therapies with faster response after 6 months as compared with CTX-based therapies, while no significant difference between TAC-based therapies and CTX-based therapies for faster response after 12 months [33]. Zhu et al. conducted a meta-analysis of four RCTs and two prospective cohort studies and found similar therapeutic effects between TAC and CTX for inducing renal remission within 1 year. Moreover, TAC was associated with an increased risk of tremor, and reduced risk of leukopenia [34]. A meta-analysis conducted by Lin et al. [35] found TAC therapy was associated with high total remission and low proteinuria level as compared with CTX for patients with IMN. However, several new studies met the inclusion criteria should be entered into meta-analysis. Moreover, whether the treatment effectiveness between groups is differing according to follow-up duration were also inconclusive. Therefore, we conducted an updated meta-analysis of RCTs to compare the therapeutic effects between TAC and CTX for IMN patients undergoing steroids.

The summary results of this study found TAC were associated with higher incidence of overall remission and complete remission than CTX for IMN patients, especially for patients after 6 months follow-up. Mostly included studies reported similar conclusions, whereas several studies did not find a significant difference between TAC and CTX. The reason for this could be the sample size from included studies was not enough to detect significant difference between TAC and CTX. However, the study conducted by Ramachandran et al. [17] found the incidence of overall remission in TAC group was lower than CTX group after 12 months follow-up. The potential reason for this could be steroids was withdrawal after 6 months. Moreover, the significant differences between TAC and CTX for the incidences of overall remission and complete remission could explained by the following reasons: (1) TAC as an inhibitor of calcium enzyme could combined with immune avidin and suppress cytoplasm calcineurin activity, then block the T cell proliferation and not associated with bone marrow suppression; (2) the combined use of TAC and steroids could inhibits the activity and proliferation of T cells *in vivo*, and reduce the expression of CnAa. The combined use TAC and steroids could block the transcription of T lymphocyte and affecting the antibody of B lymphocytes, then improving the prognosis of IMN.

We noted TAC therapy was associated with lower urinary protein excretion, irrespective after 6 or 12 months follow-up. Mostly included studies reported similar conclusions, and only two of included studies did not found significant difference between TAC and CTX [13,16]. The potential reason for this could be the improving symptom for IMN patients treated with TAC plus steroids. Moreover, we noted TAC was associated with higher serum albumin than CTX, and all of studies reported a similar trend. Finally, there was no significant difference between TAC and CTX for serum creatinine for IMN patients, and only one study found TAC was associated with lower serum creatinine than CTX [21]. Therefore, the renal function of IMN patients treated with TAC or CTX needed further large-scale RCT verified.

Subgroup analysis for the therapeutic effects between TAC and CTX were just performed according to follow-up duration. However, several other important variables in patients need addressed. Wang et al. found high expressed miR-193a, and low expressed Wilms tumor type 1 or podocalyxin was associated with poor prognosis for IMN, including elevated proteinuria or serum creatinine, and reduced glomerular filtration rate [36]. Moreover, the baseline proteinuria level in IMN patients is significantly associated with the prognosis, and high proteinuria level with poor prognosis for IMN patients [37]. Finally, the results of this study were restricted in a resource-limited environments, and additional novel treatment strategies should be further explored for improving the prognosis of IMN.

As expected, the adverse events were more obvious for patients in CTX group. The summary results found TAC was associated with a reduced risk of alopecia, infection, leukocytosis, and elevated ALT/AST, while it could increase the risk of tremor. The potential reason for this could be the effectiveness of CTX was unstable, and patients presented with severe infection, bone marrow or gonad inhibition are significantly associated with the absorption and metabolism of CTX. The use of TAC could yield beneficial effect on glomerulonephritis, it also could effective against autoimmune diseases. Therefore, the safety of TAC was superior than CTX for patients with IMN.

The strengths of this study should be highlighted: (1) the analysis of this study based on published RCTs, and the pooled conclusions based on high evidence level; (2) the analysis of this study based on large sample size, and the conclusions are more robust than individual trial; (3) this study given comprehensive results for the incidences of overall remission, complete remission, and renal function parameters; and (4) subgroup analyses for investigated outcomes based on follow-up duration were also performed.

Although above, several limitations of this study should be acknowledged. First, the quality of several included studies was low to moderate, which could affect the reliable of pooled conclusions. Second, the heterogeneity across included studies for several outcomes was not fully explained by using sensitivity and subgroup analyses. Third, all of the included studies were conducted in China or India, and the generalizability for pooled conclusion was restricted. Forth, the transparency of this study was restricted owing to the protocol was not registered; Fifth, the analysis of this study focused on intermediate endpoints, whereas the long-term effectiveness between TAC and CTX for IMN were not illustrated; Sixth, publication bias was inevitable owing the analysis based on published articles. Finally, the analysis of this study on the basis of pooled data, and the details analyses were restricted.

In summary, the findings of this study found TAC plus steroids given a better therapeutic effect on overall remission, complete remission, urinary protein excretion, and higher serum albumin as compared with CTX plus steroids. However, no significant difference between TAC and CTX for serum creatinine was detected for IMN patients. Moreover, the better therapeutic effects of TAC plus steroids mainly detected after 6 months follow-up. Furthermore, we noted the safety profile for patients in TAC group was superior than those in CTX group. Further high-quality RCT should be conducted to assess the long-term prognosis of IMN patients treated with TAC plus steroids.

## Supplementary Material

Supplemental Material

Supplemental Material

Supplemental Material

Supplemental Material

## Data Availability

The authors confirm that the data supporting the findings of this study are available within the article.
