# Supplementary material for: Tacrolimus versus cyclophosphamide for patients with idiopathic membranous nephropathy and treated with steroids: a systematic review and meta-analysis of randomized controlled trials
Source: Ren Fail. 2021 May 21;43(1):840–50. doi: 10.1080/0886022X.2021.1914655 (PMC8158268; doi:10.1080/0886022X.2021.1914655)
Supplement: Supplemental Material [file IRNF_A_1914655_SM1327.pdf]

Search strategy in PubMed:

1. ("Tacrolimus/administration and dosage"[Mesh] OR "Tacrolimus/adverse effects"[Mesh] OR "Tacrolimus/therapeutic use"[Mesh] OR "Tacrolimus/toxicity"[Mesh])

2. FK506

3. TAC

4. OR/1-3

5. ("Glomerulonephritis, Membranous/drug therapy"[Mesh] OR "Glomerulonephritis, Membranous/therapy"[Mesh])

6. idiopathic membranous nephropathy

7. membranous nephropathy

8. membranous glomerulonephropathy

9. IMN

10. MN

11. OR/5-10

12. 4 AND 11

Search strategy in EmBase:

('tacrolimus'/exp OR 'FK506' OR 'TAC') AND ('idiopathic membranous nephropathy'/exp OR 'membranous nephropathy' OR 'membranous glomerulonephropathy' OR 'IMN' OR 'MN')
