# Supplementary material for: Tacrolimus versus cyclophosphamide for patients with idiopathic membranous nephropathy and treated with steroids: a systematic review and meta-analysis of randomized controlled trials
Source: Ren Fail. 2021 May 21;43(1):840–50. doi: 10.1080/0886022X.2021.1914655 (PMC8158268; doi:10.1080/0886022X.2021.1914655)
Supplement: Supplemental Material [file IRNF_A_1914655_SM1326.pdf]

Excluding articles:

(1) no appropriate control:

1. Chen W, Liu Q, Liao Y, Yang Z, Chen J, Fu J, Zhang J, Kong Y, Fu P, Lou T, Liu Z, Ji Y, Li Z, Yu X. Outcomes of tacrolimus therapy in adults with refractory membranous nephrotic syndrome: a prospective, multicenter clinical trial. Am J Med Sci. 2013 Feb;345(2):81-7.

2. Yuan H, Liu N, Sun GD, Jia Y, Luo P, Miao LN. Effect of prolonged tacrolimus treatment in idiopathic membranous nephropathy with nephrotic syndrome. Pharmacology. 2013;91(5-6): 259-66.

3. Ballarin J, Poveda R, Ara J, Pérez L, Calero F, Grinyó JM, Romero R. Treatment of idiopathic membranous nephropathy with the combination of steroids, tacrolimus and mycophenolate mofetil: results of a pilot study. Nephrol Dial Transplant. 2007 Nov;22(11):3196-201.

4. Cosio FG, Amer H, Grande JP, Larson TS, Stegall MD, Griffin MD. Comparison of low versus high tacrolimus levels in kidney transplantation: assessment of efficacy by protocol biopsies. Transplantation. 2007 Feb 27;83(4):411-6.

5. Kempers S, Boguniewicz M, Carter E, Jarratt M, Pariser D, Stewart D, Stiller M, Tschen E, Chon K, Wisseh S, Abrams B. A randomized investigator-blinded study comparing pimecrolimus cream 1% with tacrolimus ointment 0.03% in the treatment of pediatric patients with moderate atopic dermatitis. J Am Acad Dermatol. 2004 Oct;51(4):515-25.

6. Canzanello VJ, Textor SC, Taler SJ, Schwartz LL, Porayko MK, Wiesner RH, Krom RA. Late hypertension after liver transplantation: a comparison of cyclosporine and tacrolimus (FK 506). Liver Transpl Surg. 1998 Jul;4(4):328-34

7. Wiesner RH. Long-term comparison of tacrolimus versus cyclosporine in liver transplantation. The US FK Study Group. Transplant Proc. 1998 Jun;30(4):1399-400.

8. Canzanello VJ, Schwartz L, Taler SJ, Textor SC, Wiesner RH, Porayko MK, Krom RA. Evolution of cardiovascular risk after liver transplantation: a comparison of cyclosporine A and tacrolimus (FK506). Liver Transpl Surg. 1997 Jan;3(1):1-9.

9. Porayko MK, Gonwa TA, Klintmalm GB, Wiesner RH. Comparing nephrotoxicity of FK 506 and cyclosporine regimens after liver transplantation: preliminary results from US Multicenter trial. U.S. Multicenter Liver Study Group. Transplant Proc. 1995 Feb;27(1):1114-6.

10. Porayko MK, Gonwa TA, Klintmalm GB, Wiesner RH. Comparing nephrotoxicity of FK 506 and cyclosporine regimens after liver transplantation: preliminary results from US Multicenter trial. U.S. Multicenter Liver Study Group. Transplant Proc. 1995 Feb;27(1):1114-6.

11. Porayko MK, Textor SC, Krom RA, Hay JE, Gores GJ, Wahlstrom HE, Sanchez-Urdazpal L, Richards T, Crotty P, Beaver S, et al. Nephrotoxicity of FK 506 and cyclosporine when used as primary immunosuppression in liver transplant recipients. Transplant Proc. 1993 Feb;25(1 Pt 1):665-8.

12. Charlton M, Rinella M, Patel D, McCague K, Heimbach J, Watt K. Everolimus Is Associated With Less Weight Gain Than Tacrolimus 2 Years After Liver Transplantation: Results of a Randomized Multicenter Study. Transplantation. 2017 Dec;101(12):2873-2882.

13. Qin HZ, Liu L, Liang SS, Shi JS, Zheng CX, Hou Q, Lu YH, Le WB. Evaluating tacrolimus treatment in idiopathic membranous nephropathy in a cohort of 408 patients. BMC Nephrol. 2017 Jan 5;18(1):2.

14. Israni AK, Riad SM, Leduc R, Oetting WS, Guan W, Schladt D, Matas AJ, Jacobson PA; DeKAF Genomics Investigators. Tacrolimus trough levels after month 3 as a predictor of acute rejection following kidney transplantation: a lesson learned from DeKAF Genomics. Transpl Int. 2013 Oct;26(10):982-9.

15. Wlodarczyk Z, Vanrenterghem Y, Krämer BK, Squifflet JP, Ostrowski M. A multicenter, randomized, double-blind study comparing different FK778 doses (manitimus) with tacrolimus and steroids vs. MMF with tacrolimus and steroids in renal transplantation. BMC Nephrol. 2012 Jul 26;13:68.

16. Larson TS, Dean PG, Stegall MD, Griffin MD, Textor SC, Schwab TR, Gloor JM, Cosio FG, Lund WJ, Kremers WK, Nyberg SL, Ishitani MB, Prieto M, Velosa JA. Complete avoidance of calcineurin inhibitors in renal transplantation: a randomized trial comparing sirolimus and

tacrolimus. Am J Transplant. 2006 Mar;6(3):514-22.

(2) did not combined with steroids:

1. Nikolopoulou A, Condon M, Turner-Stokes T, Cook HT, Duncan N, Galliford JW, Levy JB, Lightstone L, Pusey CD, Roufosse C, Cairns TD, Griffith ME. Mycophenolate mofetil and tacrolimus versus tacrolimus alone for the treatment of idiopathic membranous glomerulonephritis: a randomised controlled trial. BMC Nephrol. 2019 Sep 6;20(1):352.

2. Liang Q, Li H, Xie X, Qu F, Li X, Chen J. The efficacy and safety of tacrolimus monotherapy in adult-onset nephrotic syndrome caused by idiopathic membranous nephropathy. Ren Fail. 2017 Nov;39(1):512-518.

3. Sun Z, Ren M, Wu Q, Du X. Co-administration of Wuzhi capsules and tacrolimus in patients with idiopathic membranous nephropathy: clinical efficacy and pharmacoeconomics. Int Urol Nephrol. 2014 Oct;46(10):1977-82.

4. Asrani SK, Wiesner RH, Trotter JF, Klintmalm G, Katz E, Maller E, Roberts J, Kneteman N, Teperman L, Fung JJ, Millis JM. De novo sirolimus and reduced-dose tacrolimus versus standard-dose tacrolimus after liver transplantation: the 2000-2003 phase II prospective randomized trial. Am J Transplant. 2014 Feb;14(2):356-66.

5. Praga M, Barrio V, Juárez GF, Luño J; Grupo Español de Estudio de la Nefropatía Membranosa. Tacrolimus monotherapy in membranous nephropathy: a randomized controlled trial. Kidney Int. 2007 May;71(9):924-30.

6. Bolaños-Meade J, Reshef R, Fraser R, Fei M, Abhyankar S, Al-Kadhimi Z, Alousi AM, Antin JH, Arai S, Bickett K, Chen YB, Damon LE, Efebera YA, Geller NL, Giralt SA, Hari P, Holtan SG, Horowitz MM, Jacobsohn DA, Jones RJ, Liesveld JL, Logan BR, MacMillan ML, Mielcarek M, Noel P, Pidala J, Porter DL, Pusic I, Sobecks R, Solomon SR, Weisdorf DJ, Wu J, Pasquini MC, Koreth J. Three prophylaxis regimens (tacrolimus, mycophenolate mofetil, and cyclophosphamide; tacrolimus, methotrexate, and bortezomib; or tacrolimus, methotrexate, and maraviroc) versus tacrolimus and methotrexate for prevention of graft-versus-host disease with haemopoietic cell transplantation with reduced-intensity conditioning: a randomised phase 2 trial with a

non-randomised contemporaneous control group (BMT CTN 1203). Lancet Haematol. 2019 Mar;6(3):e132-e143.

7. Koreth J, Kim HT, Lange PB, Poryanda SJ, Reynolds CG, Rai SC, Armand P, Cutler CS, Ho VT, Glotzbecker B, Yusuf R, Nikiforow S, Chen YB, Dey B, McMasters M, Ritz J, Blazar BR, Soiffer RJ, Antin JH, Alyea EP 3rd. Bortezomib-based immunosuppression after reduced-intensity conditioning hematopoietic stem cell transplantation: randomized phase II results. Haematologica. 2018 Mar;103(3):522-530.

8. Cutler C, Logan B, Nakamura R, Johnston L, Choi S, Porter D, Hogan WJ, Pasquini M, MacMillan ML, Hsu JW, Waller EK, Grupp S, McCarthy P, Wu J, Hu ZH, Carter SL, Horowitz MM, Antin JH. Tacrolimus/sirolimus vs tacrolimus/methotrexate as GVHD prophylaxis after matched, related donor allogeneic HCT. Blood. 2014 Aug 21;124(8):1372-7.

9. Segarra-Medrano A, Jatem-Escalante E, Carnicer-Cáceres C, Agraz-Pamplona I, Salcedo MT, Valtierra N, Ostos-Roldán E, Arredondo KV, Jaramillo J. Evolution of antibody titre against the M-type phospholipase A2 receptor and clinical response in idiopathic membranous nephropathy patients treated with tacrolimus. Nefrologia. 2014;34(4):491-7.

10. Dean PG, Lund WJ, Larson TS, Prieto M, Nyberg SL, Ishitani MB, Kremers WK, Stegall MD. Wound-healing complications after kidney transplantation: a prospective, randomized comparison of sirolimus and tacrolimus. Transplantation. 2004 May 27;77(10):1555-61.

11. Dorr CR, Wu B, Remmel RP, Muthusamy A, Schladt DP, Abrahante JE, Guan W, Mannon RB, Matas AJ, Oetting WS, Jacobson PA, Israni AK; for DeKAF Genomics. Identification of genetic variants associated with tacrolimus metabolism in kidney transplant recipients by extreme phenotype sampling and next generation sequencing. Pharmacogenomics J. 2019 Aug;19(4):375-389.

12. Sanghavi K, Brundage RC, Miller MB, Schladt DP, Israni AK, Guan W, Oetting WS, Mannon RB, Remmel RP, Matas AJ, Jacobson PA. Genotype-guided tacrolimus dosing in African-American kidney transplant recipients. Pharmacogenomics J. 2017 Jan;17(1):61-68.

13. Suszynski TM, Gillingham KJ, Rizzari MD, Dunn TB, Payne WD, Chinnakotla S, Finger EB,

Sutherland DER, Najarian JS, Pruett TL, Matas AJ, Kandaswamy R. Prospective randomized trial of maintenance immunosuppression with rapid discontinuation of prednisone in adult kidney transplantation. Am J Transplant. 2013 Apr;13(4):961-970.

(3) not RCTs

1. Liu S, Li X, Li H, Liang Q, Chen J, Chen J. Comparison of tripterygium wilfordii multiglycosides and tacrolimus in the treatment of idiopathic membranous nephropathy: a prospective cohort study. BMC Nephrol. 2015 Dec 4;16:200.

2. Li X, Lv R, He Q, Li H, Du X, Lin W, Li Q, He X, Wang S, Chen J. Early initiation of tacrolimus or cyclophosphamide therapy for idiopathic membranous nephropathy with severe proteinuria. J Nephrol. 2008 Jul-Aug;21(4):584-91.

3. Caro J, Gutiérrez-Solís E, Rojas-Rivera J, Agraz I, Ramos N, Rabasco C, Espinosa M, Valera A, Martín M, Frutos MÁ, Perea L, Juárez GF, Ocaña J, Arroyo D, Goicoechea M, Fernández L, Oliet A, Hernández Y, Romera A, Segarra A, Praga M; Grupo de Estudio de las Enfermedades Glomerulares de la Sociedad Española de Nefrología (GLOSEN). Predictors of response and relapse in patients with idiopathic membranous nephropathy treated with tacrolimus. Nephrol Dial Transplant. 2015 Mar;30(3):467-74.

4. Watt KD, Dierkhising R, Heimbach JK, Charlton MR. Impact of sirolimus and tacrolimus on mortality and graft loss in liver transplant recipients with or without hepatitis C virus: an analysis of the Scientific Registry of Transplant Recipients Database. Liver Transpl. 2012 Sep;18(9):1029-36.

5. Bongartz T, Ryu JH, Matteson EL. Is tacrolimus effective for treating antisynthetase-associated interstitial lung disease? Nat Clin Pract Rheumatol. 2005 Dec;1(2):80-1.

6. Shang SL, Cai GY, Duan SW, Li P, Li QG, Chen XM. Retrospective analysis of tacrolimus combined with Tripterygium wilfordii polyglycoside for treating idiopathic membranous nephropathy. BMC Nephrol. 2018 Jul 18;19(1):182.

7. Kumar V, Varma AK, Nada R, Ghosh R, Suri D, Gupta A, Kumar V, Rathi M, Kohli H, Jha V, Gupta K, Ramachandran R. Primary membranous nephropathy in adolescence: A prospective

study. Nephrology (Carlton). 2017 Sep;22(9):678-683.

(4) review or meta-analysis

1. Zheng Q, Yang H, Liu W, Sun W, Zhao Q, Zhang X, Jin H, Sun L. Comparative efficacy of 13 immunosuppressive agents for idiopathic membranous nephropathy in adults with nephrotic syndrome: a systematic review and network meta-analysis. BMJ Open. 2019 Sep 11;9(9):e030919.

2. Lin W, Li HY, Lin S, Zhou T. Efficacy and safety of tacrolimus vs cyclophosphamide in the therapy of patients with idiopathic membranous nephropathy: a meta-analysis. Drug Des Devel Ther. 2019 Jul 3;13:2179-2186.

3. Liu D, Yang Y, Kuang F, Qing S, Hu B, Yu X. Risk of infection with different immunosuppressive drugs combined with glucocorticoids for the treatment of idiopathic membranous nephropathy: A pairwise and network meta-analysis. Int Immunopharmacol. 2019 May;70:354-361.

4. Li YC, Huang J, Li X, Zhao SM. A comparison of cyclophosphamide versus tacrolimus in terms of treatment effect for idiopathic membranous nephropathy: A meta-analysis. Nefrologia. 2019 May-Jun;39(3):269-276.
