# Supplementary material for: Tacrolimus versus cyclophosphamide for patients with idiopathic membranous nephropathy and treated with steroids: a systematic review and meta-analysis of randomized controlled trials
Source: Ren Fail. 2021 May 21;43(1):840–50. doi: 10.1080/0886022X.2021.1914655 (PMC8158268; doi:10.1080/0886022X.2021.1914655)
Supplement: Supplemental Material [file IRNF_A_1914655_SM1325.pdf]

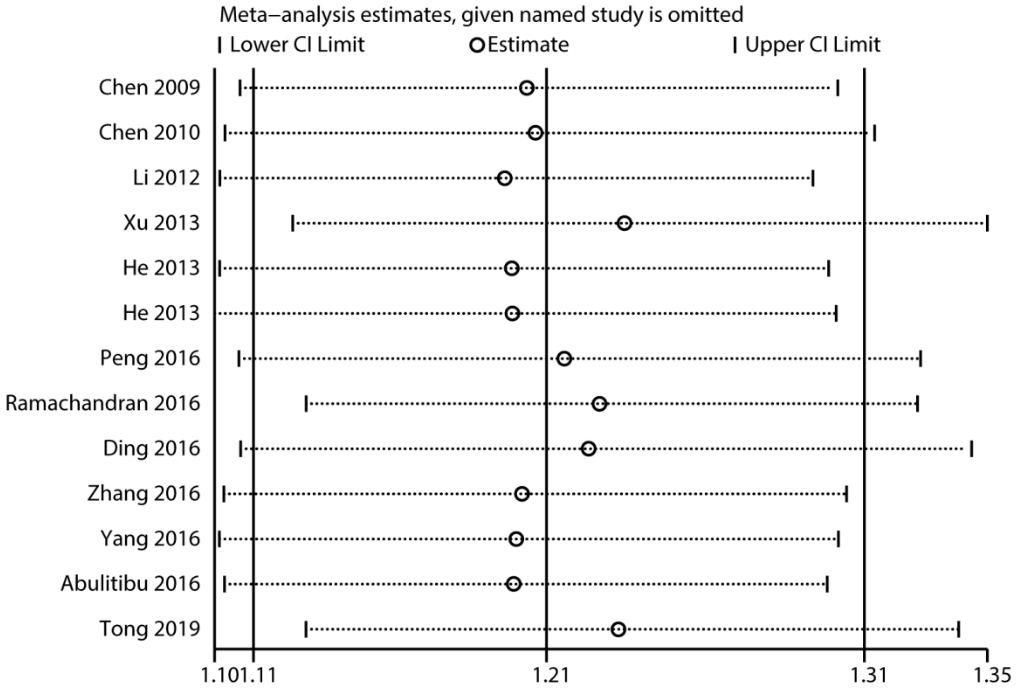

Figure S1. Sensitivity analysis for overall remission

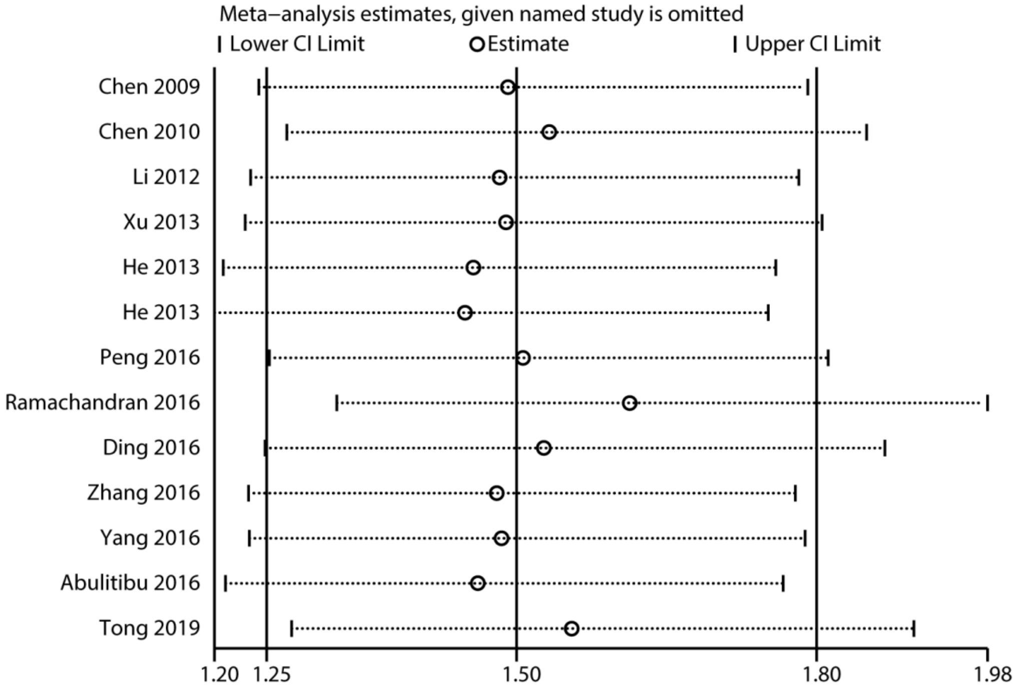

Figure S2. Sensitivity analysis for complete remission

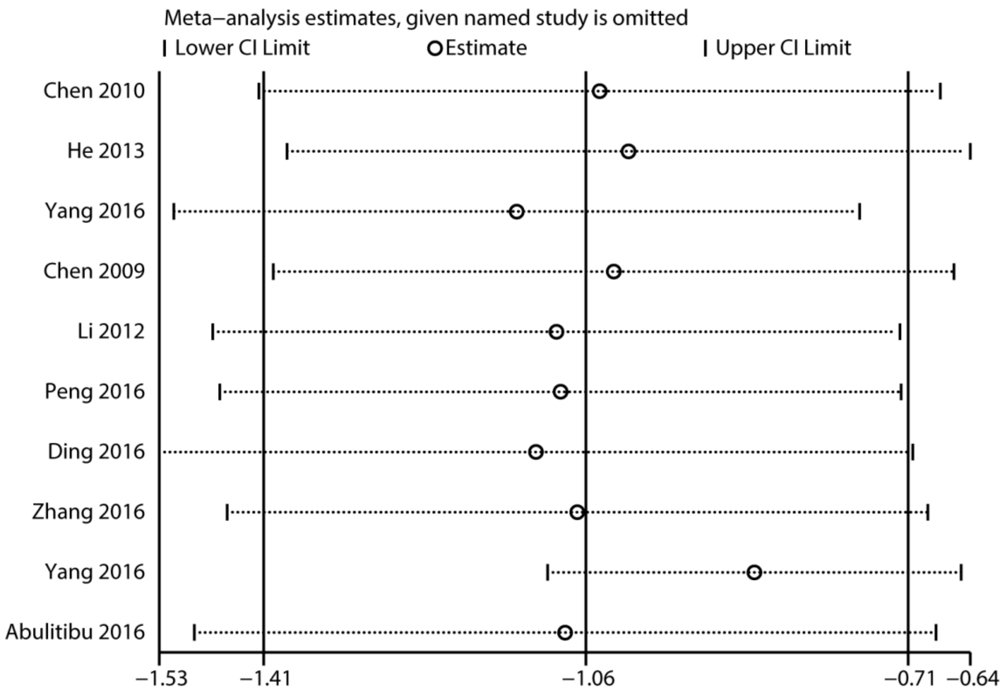

Figure S3. Sensitivity analysis for urinary protein excretion

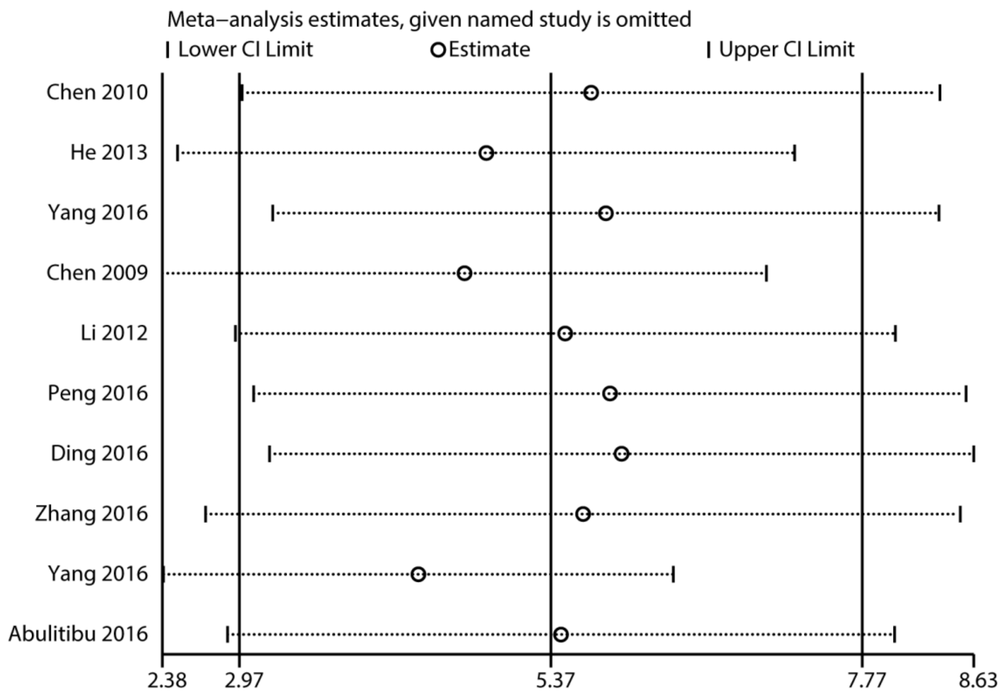

Figure S4. Sensitivity analysis for serum albumin

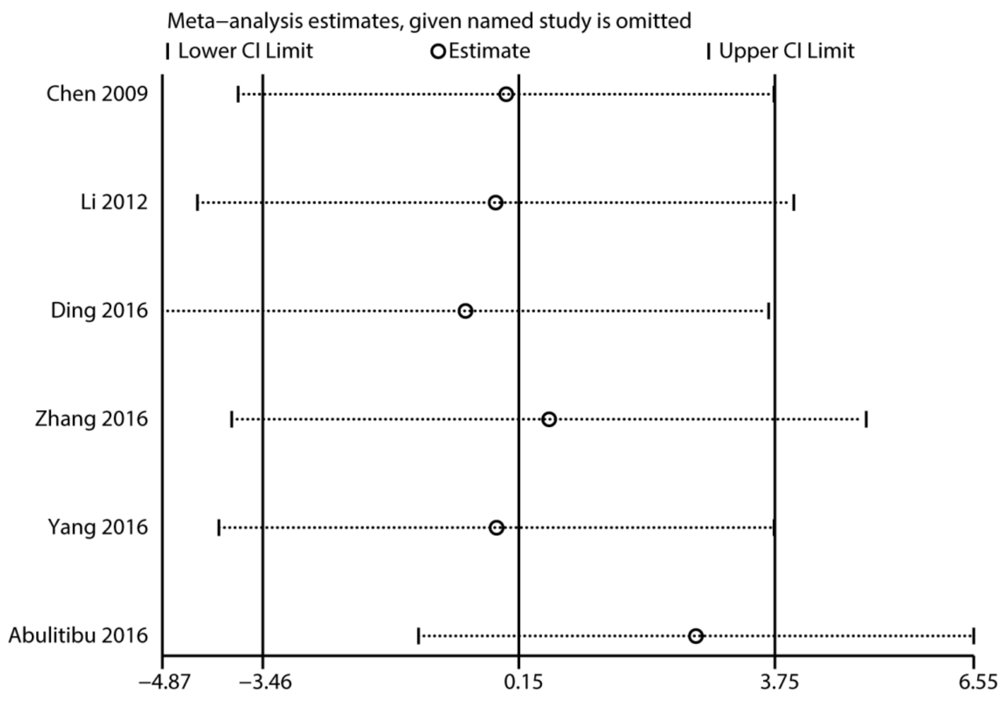

Figure S5. Sensitivity analysis for serum creatinine
