# Supplementary material for: Tacrolimus versus cyclophosphamide for patients with idiopathic membranous nephropathy and treated with steroids: a systematic review and meta-analysis of randomized controlled trials
Source: Ren Fail. 2021 May 21;43(1):840–50. doi: 10.1080/0886022X.2021.1914655 (PMC8158268; doi:10.1080/0886022X.2021.1914655)
Supplement: Supplemental Material [file IRNF_A_1914655_SM1322.pdf]

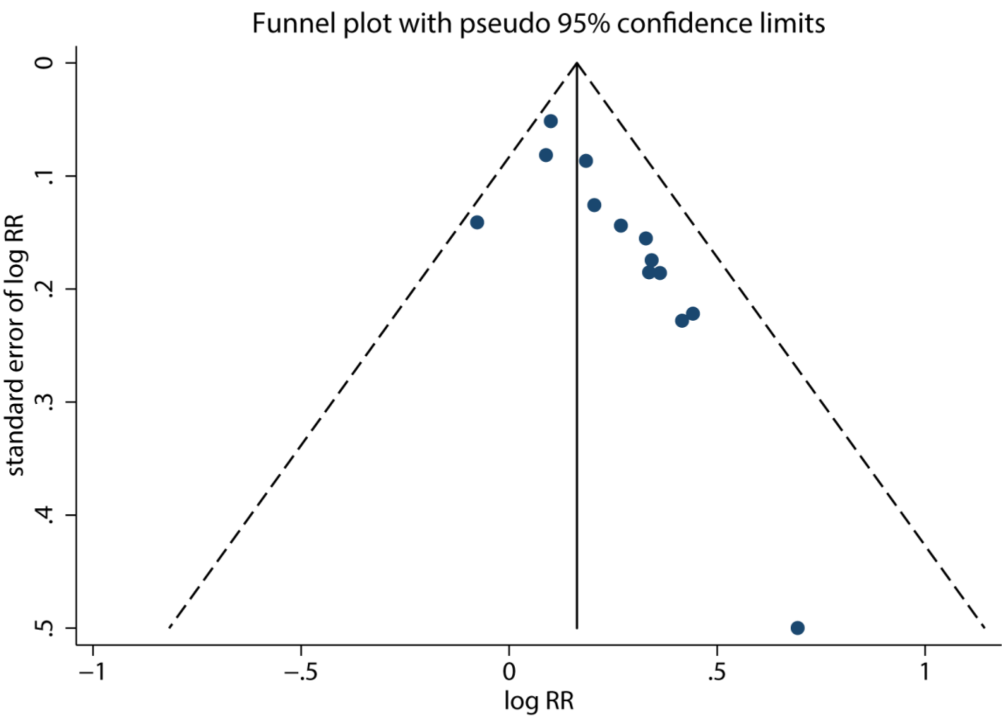

P value for Egger: 0.003 P value for Begg: 0.001

Figure S1. Funnel plot for overall remission

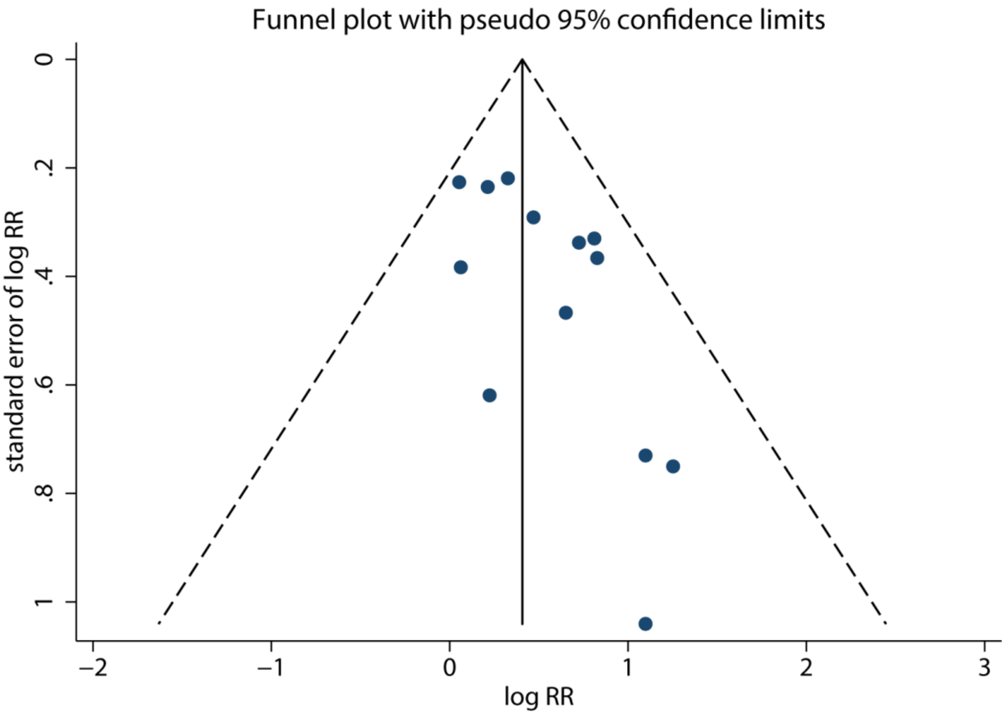

P value for Egger: 0.027 P value for Begg: 0.200

Figure S2. Funnel plot for complete remission

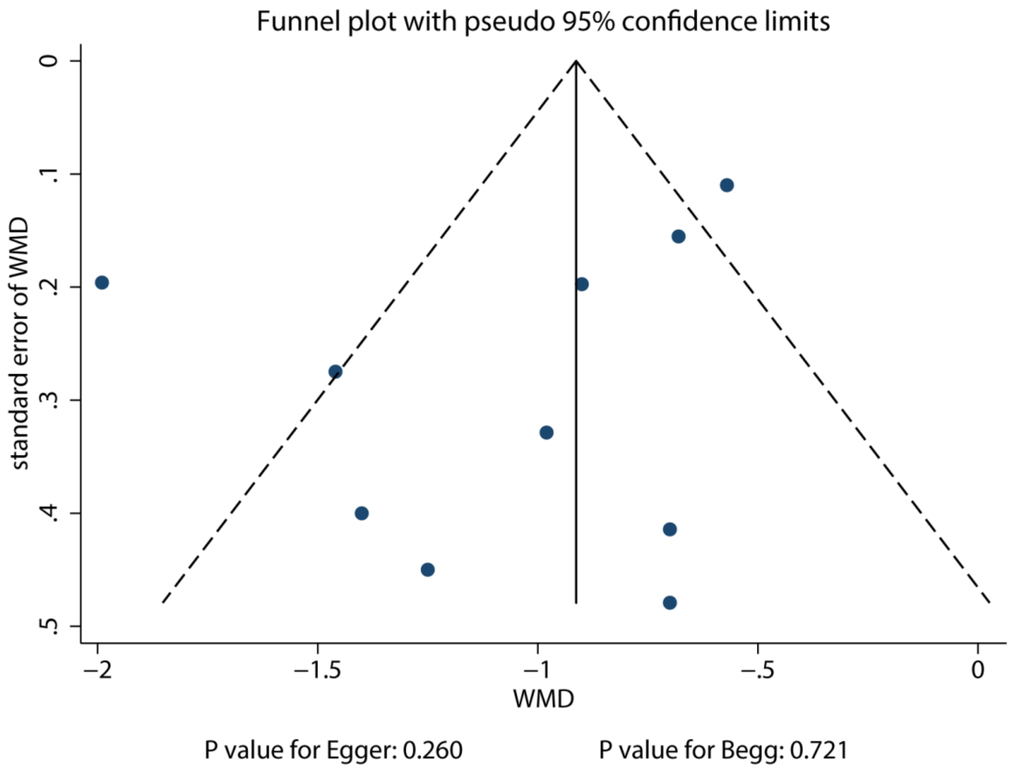

Figure S3. Funnel plot for urinary protein excretion

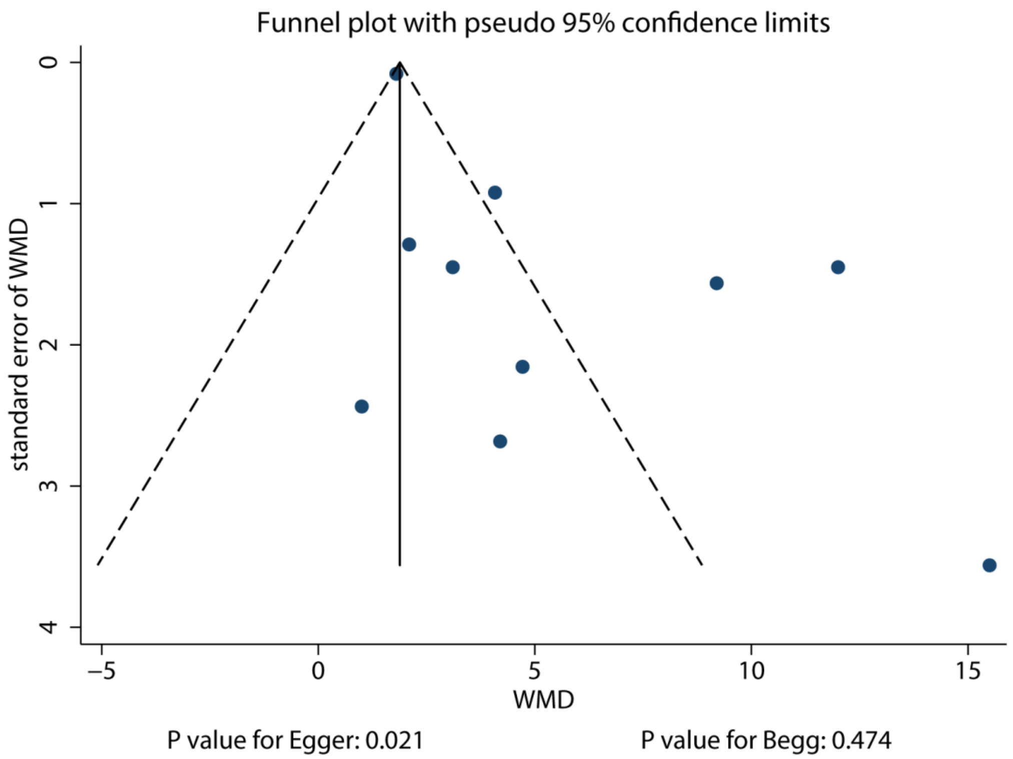

Figure S4. Funnel plot for serum albumin

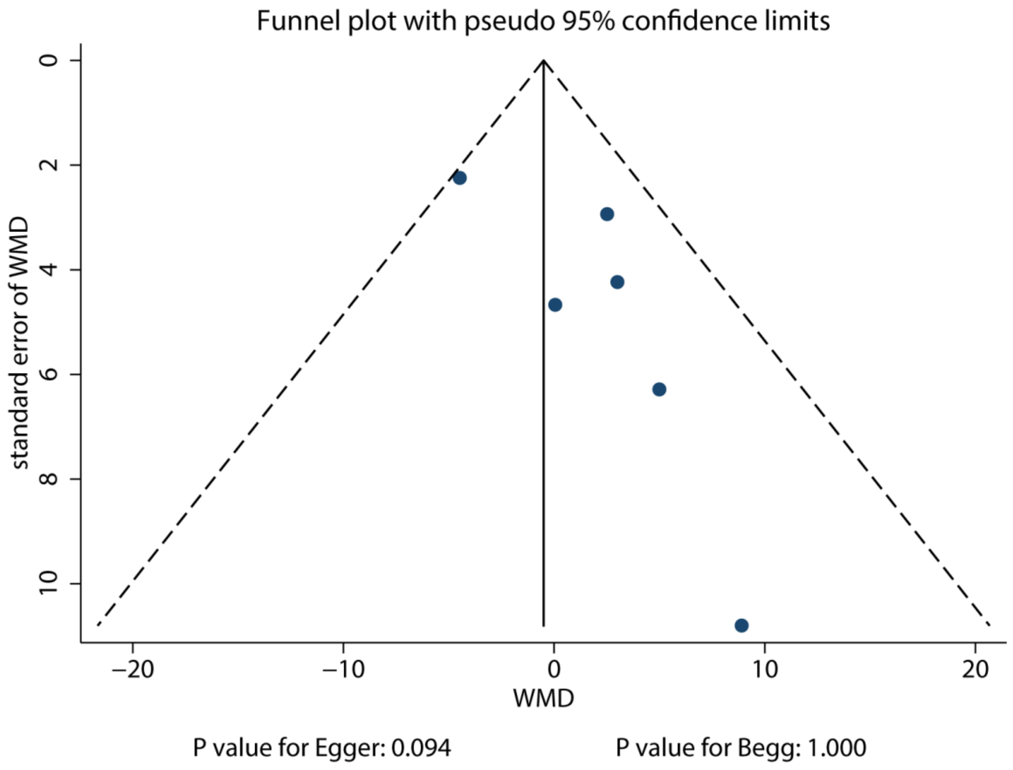

Figure S5. Funnel plot for serum creatinine
